# Some Aspects of Oxidation and Reduction Processes in Ti–Al and Ti–Al–Nb Systems

**DOI:** 10.3390/ma15051640

**Published:** 2022-02-22

**Authors:** Marzena Mitoraj-Królikowska, Ewa Drożdż

**Affiliations:** Inorganic Chemistry Department, Faculty of Materials Science and Ceramics, AGH University of Science and Technology, Al. Mickiewicza 30, 30-059 Krakow, Poland; edrozdz@agh.edu.pl

**Keywords:** titanium, titanium aluminides, TNB, oxidation, reduction

## Abstract

The oxidation of titanium and titanium aluminides has attracted the attention of scientists for many years because of their high-temperature application. The most popular method to investigate oxidation behavior is the measurement of alloy mass changes during exposure to elevated-temperature under isothermal or thermal cycling conditions. However, the thermogravimetric method is not enough to establish an oxidation mechanism. In this paper, the temperature-programmed oxidation (TPOx) and reduction (TPR) were applied for the Ti–Al and Ti–Al–Nb systems, which was a new experimental concept which revealed interesting phenomena. Although oxidation of titanium alloys is well-described in the literature, not many papers present at the same time reduction of oxidized alloys. The results presented in the paper concentrated on the first stages of oxidation, which are scarcely described in the literature, but are important to understand the oxidation mechanism. Comparison between powder and bulk samples with similar compositions revealed essential differences in the oxidation mechanism.

## 1. Introduction

There is a lot of information in the literature on the oxidation of titanium and titanium aluminides alloys at various temperatures. Generally, the titanium oxidation reaction is described as the result of two processes: diffusion of oxygen into the bulk of the sample and desorption of O atoms from the surface with concomitant reduction of the Ti oxidation state. The diffusion process is stimulated by temperature increase, however at the same time, as the temperature is rising significantly, the process of oxygen desorption becomes more intense. Moreover, it is known that high-temperature treatment promotes the mobility of oxygen in the oxide lattice. In general, the mentioned processes are controlled mainly by the duration of exposure to O_2_ and the temperature of the samples. According to Lu et al. [1] these two opposing trends indicate 227–327 °C as the optimal temperature-range for maximum oxidation of titanium. Furthermore, titanium at room-temperature (RT) oxidizes at the very beginning of oxygen exposure, and the only oxide recognized at RT is TiO_2_. The oxidation at a higher-temperature is different and is determined by oxygen solubility [2]. The maximum solubility of oxygen in alfa titanium according to the concentration profile for the solid solution of Ti (O) is equal to 34 at.% [3] and is a function of temperature and time. Maximum solubility could be achieved in relatively short time at the temperature above 760 °C [4]. The composition of titanium compounds depends on time and temperature. The rutile phase (TiO_2_) is present in the oxidation products in all temperature-ranges [5]. At a higher-temperature, traces of other oxides, such as Ti_2_O_3_ (above 650 °C) [5], TiO, and Ti_3_O_5_ (above 800 °C) [6], were also detected. Generally, under the uniform and compact TiO_2_ layer, internal, rather loose, lower titanium oxides are present [6]. 

Hydrogen reduction of TiO_2_ is an important and promising reaction for the production of environmentally friendly titanium powder. Currently, on an industrial scale the energy-consuming Kroll method is used. Theoretically, from the thermodynamic point of view, reduction of TiO_2_ has positive and negative Gibbs Free Energy value depending on the form of hydrogen:

TiO_2(s)_ + 2H_2_ → 2H_2_O_(g)_ + Ti_(s)_   ΔG°r = 431.67 kJ (1)

TiO_2(s)_ + 4H → 2H_2_O_(g)_ + Ti_(s)_   ΔG°r = −381.44 kJ (2)

The above values were determined at temperature 25 °C on the basis of data from the NIST database [7].

The aluminum oxidation at the RT is exothermic (by 3.6 kcal/mol) and the reaction mechanism is not very simple, what was predicted on the basis of the theoretical calculations by Marshall et al. [8]. Garland [9] experimentally confirmed that the reaction of Al + O_2_ in the temperature and pressure range: 25–827 °C and 0.01–0.13 atm, respectively, proceeds by transfer of O^−^ ions in two main stages: formation of the OAlO complex and decomposition of this complex to AlO + O. These stages are rarely mentioned by the authors and most of them stated that the reaction of aluminum with oxygen below 400 °C does not proceed to the crystalline phase (only a thin amorphous layer is formed) and only above this temperature this layer crystallizes: first into γ-Al_2_O_3_ phase, next with the increase in the temperature to θ-Al_2_O_3_ and then α-Al_2_O_3_ phase appears [10]. 

The idea of Al_2_O_3_ reduction with hydrogen is not very new but the reduction mechanism is still not well recognized. Similarly, like for TiO_2_, Gibbs Free Energy is only negative for atomic hydrogen:

Al_2_O_3(s)_ + 3H_2(g)_ ⇆ 2Al_(s)_ + 3H_2_O_(g)_   ΔG°r = 896.50 kJ (3)

Al_2_O_3(s)_ + 6H_(g)_ ⇆ 2Al_(s)_ + 3H_2_O_(g)_   ΔG°r = −323.16 kJ (4)

The above values were determined at temperature 25 °C on the basis of data from the NIST database [7].

Niobium has a high affinity for oxygen and in the niobium–oxygen system, the niobium element can be found in four different oxidation states: 0, 2^+^, 4^+^, and 5^+^, generally related to the phases of metallic Nb and to the NbO, NbO_2_, and Nb_2_O_5_, respectively [11]. According to Delheusy et al. [12], it is a triple layer consisting of NbO, NbO_2_, and Nb_2_O_5_. Nico et al. [13] reported that during annealing of niobium in the air atmosphere (from room-temperature to about 1000 °C) the formation of cubic NbO in the temperature-range of 100–370 °C can be observed, around 300 °C the amorphous phase appears and again, approximately 500 °C, a crystalline phase of niobium (V) oxide is created. In addition, the first orthorhombic phase was detected, while the monoclinic phase at around 850 °C also appeared. In the same article, the authors suggested that two exothermic peaks on the DTA curve between 300 and 500 °C correspond to the oxidation of NbO (the first peak) and the crystallization of Nb_2_O_5_ from amorphous phase (the second one). 

The reduction of Nb_2_O_5_ reduction is a basic process in niobium production. The most of niobium obtained from the reduction of niobium pentoxide is produced with aluminothermic reaction:

3Nb_2_O_5(s)_ + 10Al_(s)_ = 6Nb_(s)_ + 5Al_2_O_3(s)_ (5)

Sometimes the carbothermic two-stage process is used for niobium production with the overall reduction reaction:

Nb_2_O_5(s)_ + 5C_(s)_ = 2Nb_(s)_ + 5CO_(g)_ (6)

Reduction of Nb_2_O_5_ with hydrogen is rarely described in the literature. According to the basic experiments [14], niobium pentoxide was readily reduced to tetroxide, but further reduction was rather slow. 

The use of titanium alloys that contain aluminum and other alloying elements in the automotive and transport industries is often due to the strict requirements related to the high oxidation resistance of materials for such applications. As a result of high-temperature oxidation of these alloys, mainly the formation of Al_2_O_3_ and TiO_2_ oxides is observed. According to the binary Ti–Al phase diagram [15] when the concentration of aluminum in titanium is greater than 20 at.% formation of titanium aluminides intermetallic phases is observed. Titanium aluminides phases were predicted as a lightweight and oxidation resistant alloys for special applications in the automotive and airline industry. The first generation of these alloys was based on γ-TiAl phase (gamma alloy). Because of the high aluminum concentration, the oxidation resistance of the gamma alloy was better than that of titanium. However, depending on the oxygen pressure and metal activity, either Ti or Al may oxidize preferentially. The products of oxidation are rutile (TiO_2_) and α-alumina (Al_2_O_3_). Compared to alumina, titania has a high diffusivity of oxygen and poor protectiveness that result in a reduced oxidation resistance of the γ-TiAl alloy especially at temperatures exceeding 800 °C. 

The second generation of titanium aluminides alloys was represented by advanced ternary and quaternary alloys: Ti-(45-48)Al-(1-3)Cr, Mn-(2-5)Nb, Ta, Mo in at.% [16]. The third generation of the Nb- rich alloys with general composition: Ti-(45-46)Al-(5-10)Nb-(0-0.5)B, C in at.% termed as TNB alloys were developed to improve room-temperature ductility and high-temperature creep resistance [17,18]. Titanium aluminides with 45–46% of aluminum are based on two phases: γ-TiAl (85–92 vol.%) and α_2_-Ti_3_Al (8–15 vol.%) and have mainly lamellar microstructure [19]. It was also found that Nb as a slow diffuser in the Ti–Al system influences the oxidation of these alloys; however, the exact mechanism of oxidation in the presence of Nb has not yet been found [20]. Different hypotheses are described in the literature. Some authors believe that the Nb cation with valence 5^+^, when incorporated into TiO_2_ (usually nonstochiometric with composition TiO_2−X_, where X is about 0.02) substitutes for the Ti^4+^ cation and reduces oxygen vacancies in TiO_2_ and this results in decreased oxygen diffusion and consequently reduction in the growth of TiO_2_. Another explanation of Nb influence is enhancement of Al_2_O_3_ formation [21]. It is worth mentioning that the addition of niobium can also improve the oxidation resistance of titanium alloys with a lower aluminum content [22].

The aim of this work was to compare and investigate oxidation/reduction processes in Ti–Al and Ti–Al–Nb systems (for powders and solid materials) on the basis of temperature-programmed oxidation (TPOx) and reduction (TPR) measurements completed with X-ray diffraction analysis. The work was motivated by an attempt to better understand the oxidation of the Ti–Al–Nb alloy, as well as to recognize the reduction of titanium-based materials in the context of their catalytic behavior [23] and the future application in contact with hydrogen fuel.

## 2. Materials and Methods

The powder and solid materials were used for experiment. In the case of powder samples, the following commercially available materials were used: Al—aluminum powder (99.8%, about 45 µm, SunChemical, Parsippany-Troy Hills, NJ, USA); Ti—titanium powder (>98.0%, about 150 µm, Merck, Darmstadt, Germany); Nb—niobium powder (99.8%, 45 µm, Sigma Aldrich, Saint Louis, MO, USA); and Ti–Al—titanium aluminides powder (with composition: Ti–50Al in at.%, after SHS synthesis and ball milled to the gain size about 90 µm). For comparison, five solid samples were examined: Al—aluminum (electrolytic); Ti—titanium (99.6%, Goodfellow, Huntingdon, England); Nb—(99.9%, PolAura, Zabrze, Poland); Ti–Al—titanium aluminides (with composition: Ti-50Al in at.%, arc-melted powder previously mentioned powder); and Ti–Al–Nb—TNB titanium aluminides (with composition: Ti–46Al–8Nb in at.%, produced by Access e.V., Aachen, Germany with fully lamellar microstructure). Samples in the form of cuboids and cylinders (niobium) with an area of 50–70 mm^2^ and weight of about 0.1–0.2 g before measurements were ground with emery papers up to P1200 (ISO/FEPA) and rinsed with distilled water.

TPOx and TPR measurements were conducted on ChemiSorb 2750 apparatus (Micromeritics Instrument Corporation, Norcross, GA, USA) in the flow of 2% O_2_/Ar and 5% H_2_/Ar mixtures, respectively. The samples (powders/solids) were placed in a quartz reactor and after flushing the system with helium it was heated at a rate of 10 deg/min. The masses of samples used in tests were around 0.6–0.7 g for powders and around 0.1–0.2 g for solids. The materials were first oxidized (TPOx) and then reduced (TPR). The TPR/TPOx measurements were normalized with respect to the mass of the samples (to 1 gram of sample mass), due to the fact that no materials of identical mass were used for the measurements.

Powder and solid samples after TPOx or TPR measurement were examined with a scanning electron microscope (SEM) (Nova Nano SEM 200 FEI Company, Hillsboro, OR, USA) using secondary or backscattered electron images (SEI or BEI). The chemical and phase composition of the samples were determined by energy-dispersive X-ray spectroscopy (EDS) (EDAX, AMETEK, Inc., Berwyn, IL, USA) and X-ray diffraction (XRD) (Seifert XRD7, XRD Eigenmann GmbH, Felsenweg, Germany), respectively. Some of the solid samples were selected for the preparation of metallographic cross-sections. For this purpose, the samples were embedded in epoxy resin and polished with water-based diamond suspensions (grain size: 9, 3, and 1 µm). 

## 3. Results and Discussion

### 3.1. Oxidation and Reduction of Powders

**Figure 1:**
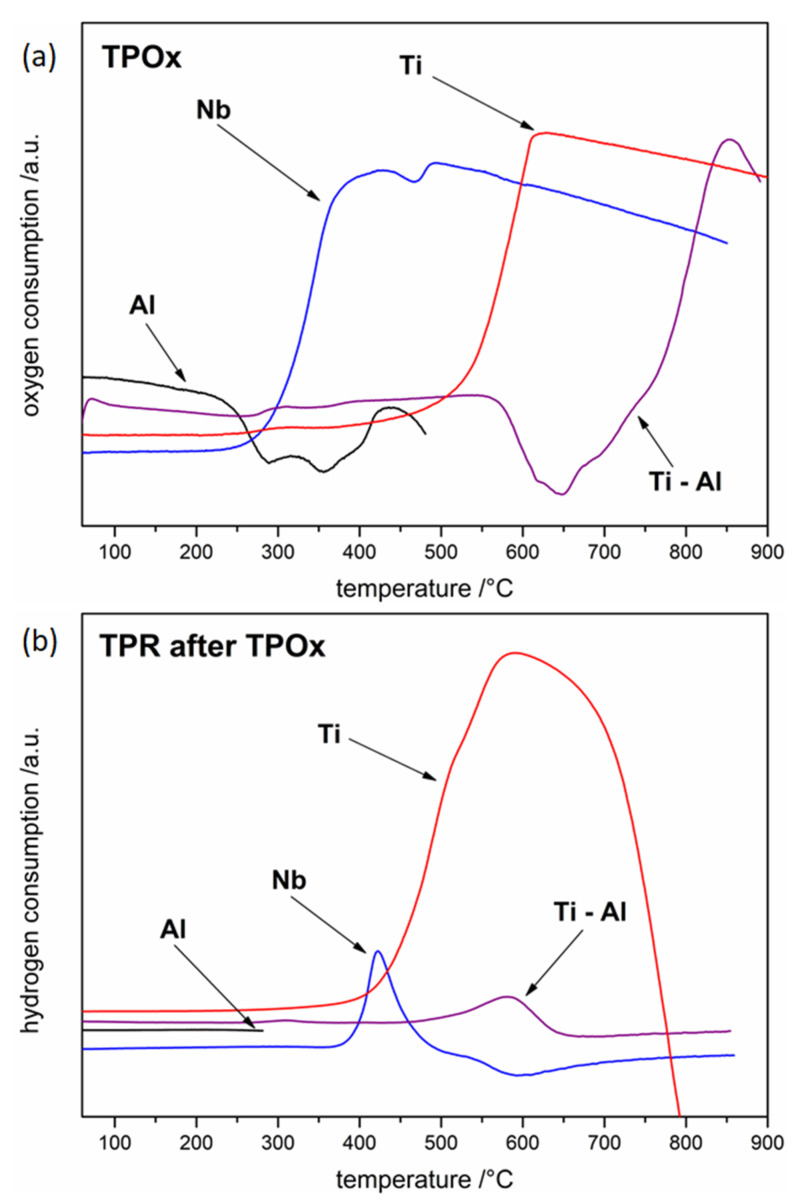
The oxidation (**a**) and reduction (**b**) profiles of tested powders.

In Figure 1, TPOx results precede TPR measurements. As can be seen in the case of the TPOx profile for Ti, a slight effect related to oxygen consumption around 230 °C appears, however only ca. 400 °C the actual process begins. During the process, the consumption of oxygen is so rapid that "oxygen starvation" is observed after a few minutes. Nevertheless, on the basis of the presented TPOx profile, it can be concluded that the oxidation of titanium is a complex process, probably associated with the formation of at least two types of oxide.

**Figure 2:**
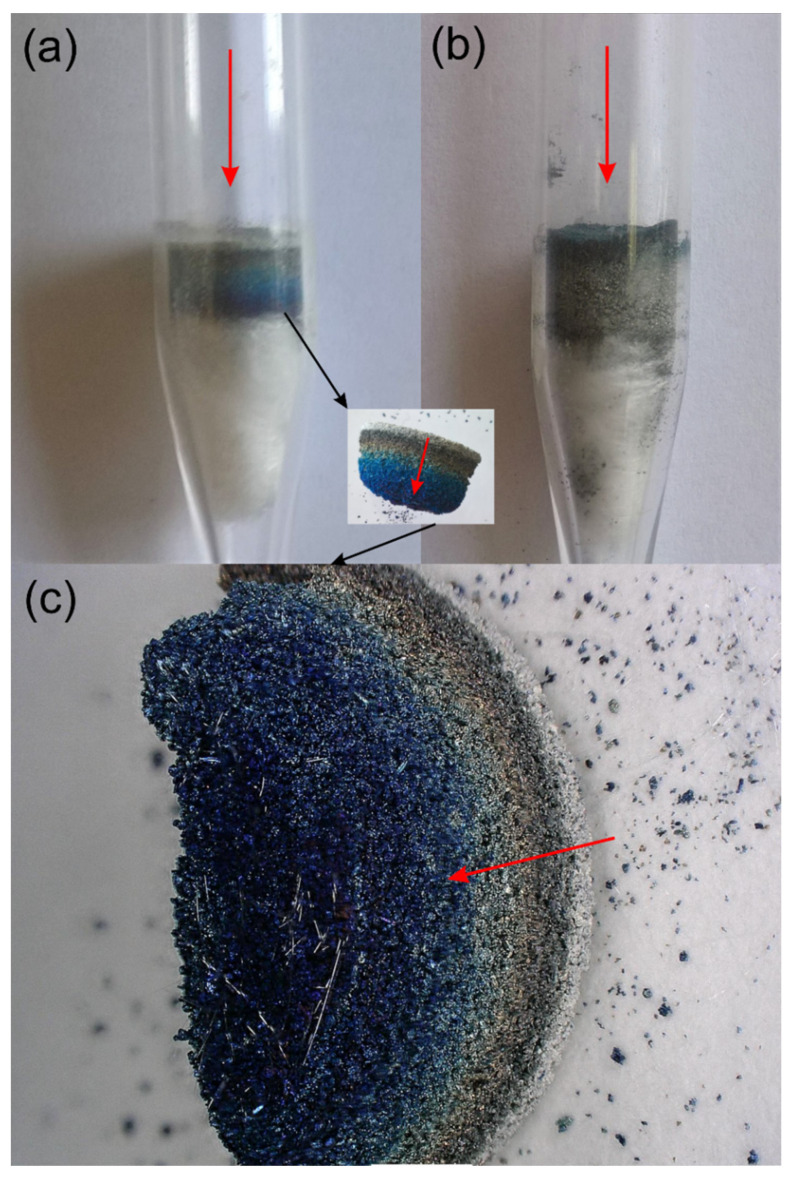
Quartz reactor with Ti powder after TPOx (**a**), and after TPOx + TPR (**b**) and a higher-magnification of oxidized powder (**c**). Arrows indicate the flow direction of the O_2_/Ar or H_2_/Ar mixture.

Inhibition of the reaction related to oxygen deficiency can additionally affect the oxidation mechanism, increasing the likelihood of the formation of titanium oxides at a lower oxidation state. Since the observations of the color of oxides are often closely related to their phase composition, the figure below shows digital pictures of the quartz reactor (used for the TPOx/TPR test) with the powder after oxidation (Figure 2a). One can notice that the colors of Ti powder after oxidation are diverse. 

**Figure 3:**
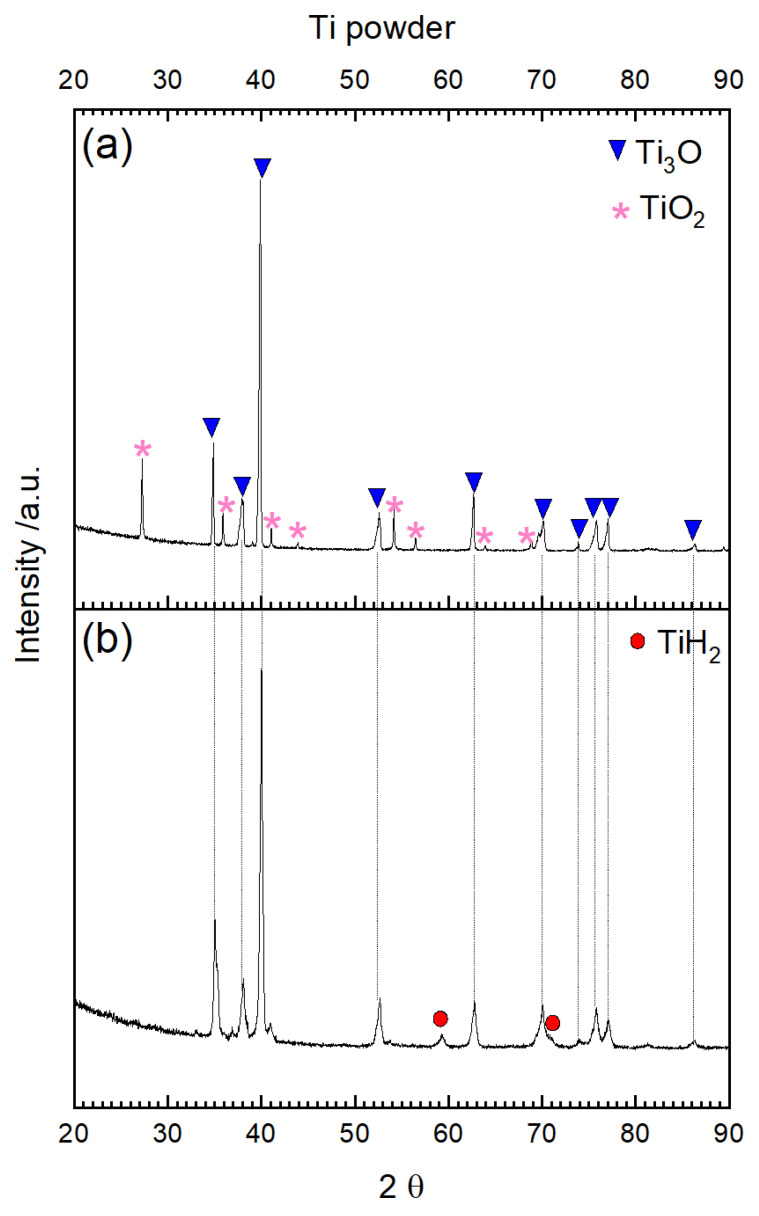
XRD diffraction pattern of Ti powder after TPOx (**a**) and after TPOx + TPR (**b**).

According to the XRD analysis shown in Figure 3a, the predominant phases for the oxidized titanium powder were Ti_3_O (hexagonal) and TiO_2_ (rutile). The titanium-richer phase (Ti_3_O) was most likely formed due to the insufficient amount of oxygen during TPOx. During oxidation, oxygen can dissolve in the octahedral interstices of titanium lattice up to 34 at.%. and occupied half of the available interstitial sites, and, therefore, the limiting composition is Ti_2_O. The Ti_3_O structure consists of a closed-packed hexagonal arrangement of titanium atoms with every second layer of octahedral interstices normal to the c-axis vacant. One-third of the oxygen sites in the occupied layers are empty and these vacancies have an ordered arrangement in the direction of the c-axis [24]. It is worth noting that the XRD results presented in Figure 3 were obtained from the powder mixture visible in the quartz reactor after oxidation (a) and after reduction (b), respectively. 

The colors visible in the reactor after TPOx in the digital picture shown in Figure 2a,c can be an effect of the presence of various oxide species, as well as TiO_2_ non-stoichiometry. It is also interesting to mention that different titanium oxides can have different colors: TiO_2_ is white, TiO has a brown-goldish color, Ti_3_O_5_ is bluish, and Ti_2_O_3_ is dark violet [25]. Different colors were also found for defective TiO_2_, for example due to the intentional introduction of oxygen vacancies as a kind of self-doping. For example, blue TiO_2_ was synthesized by incorporating Ti^3+^ derived from the TiCl_3_ precursor directly into the TiO_2_ matrix [26]. Blue hydrogenated rutile TiO_2_ was also obtained through the high-temperature and high-pressure hydrogen reduction of white rutile. The blue color of TiO_2_ is commonly ascribed to oxygen vacancy, surface disorder, and oxygen deficiency [27]. The yellow color of TiO_2_ was an effect of Nb doping by ultrasonic spray pyrolysis of the aqueous peroxide precursor solution. Nb^5+^ and Ti^3+^ ions were responsible for coloration [28]. Yellow color of TiO_2_ was also achieved without any dopants by the titanium vacancies (acceptor) and titanium interstitials (donor) that incorporated in the TiO_2_ by simple UV light assisted sol–gel method [29]. Different colors of titanium oxides were also identified during the controlled transformation of Ti_2_O_5_ to anatase/rutile TiO_2_ under oxidizing conditions. The color of the annealed particles progressively changed from black (25 °C) to dark green (500 °C), orange (600 °C), yellow (700 °C), and white (≥ 800 °C). The orange and yellow powder after annealing at 600 and 700 °C, should be the rutile TiO_2_ with oxygen vacancies [30]. The XRD analysis in Figure 3a does not confirm the presence of these oxides, suggesting that the visible colors are due to the nonstoichiometry of TiO_2_ or that the amounts of residual oxides are below the detection limit of the XRD method. 

The TPR profile for titanium (Figure 1b) indicated, that titanium is first reduced (slow onset around 250 °C); however, the actual process occurred at approximately 350 °C. The mechanism of rutile reduction was described in detail by Khader et al. [31]. According to V. Bratan et al. [32], who reported the results of the temperature-programmed reduction measurement (with heating rate of 10 deg/min in a stream of 5% H_2_/Ar), the reduction of TiO_2_ starts already above 340 °C. The TPR profile for Ti is complex (several maxima), which proves that several reduction processes occur. This can be a confirmation of the presence of several different oxide species formed during oxidation or this can be an effect of possible gradual reduction of TiO_2_ through the lower oxides. Moreover, one can notice that the TPR line at around 800 °C decreases sharply below the baseline, which indicates a rapid release of hydrogen by the system. This effect corresponds to the decomposition of titanium hydride TiH_2_, which was formed at a lower-temperature by the direct reaction of titanium with hydrogen. TiH_2_ is stable up to around 150 °C and above this temperature decomposes. It is interesting to note that reduction of TiO_2_ is described in the literature as difficult process and rather strong reducing atmospheres were used, for example hydrogen plasma [33] or metal-hydride-reduction process (MHR) [34] in order to reduce TiO_2_ only partially to Ti_3_O_5_, Ti_2_O_3_, or TiO. Theoretically, the reduction of TiO_2_ according to the phase diagram [35] should give the following oxides: TiO_2n−1_ (so-called Magneli phases), Ti_3_O_5_, Ti_2_O_3_, TiO, Ti_3_O_2_, Ti_2_O, and Ti_3_O, but rarely this sequence is followed. There is one possible explanation. Water vapor, which is a by-product of reduction reactions, would cause the reverse re-oxidation. It was also found [33] that a thinner TiO_2_ oxide is more difficult to reduce than a thicker one, which was, for example, explained with a high affinity of titanium for hydrogen. Indeed, it is well known that Ti easily reacts with hydrogen with TiH_2_ formation [36] which is another limiting factor for direct TiO_2_ to Ti reduction.

The diffraction analysis for titanium powder after TPOx–TPR cycles (Figure 3b) indicates that the reflections of the TiO_2_ phase almost disappeared, and Ti_3_O becomes the main phase. It seems that during the TPR cycle the highest Ti oxide (TiO_2_) was reduced to the lowest (Ti_3_O), possibly through the intermediate oxides, which were totally reduced at the end of TPR. At the same time, the color of the powder changed to dark gray (Figure 2b). The presence of the remaining, undecomposed TiH_2_ was also confirmed with XRD analysis (Figure 3b), which is in agreement with the described TPR curve.

Interestingly, in the case of the TPOx line for aluminum, an effect opposite to oxygen consumption was observed at a temperature of about 200 °C (Figure 1a). The complexity of the TPOx profile proves that oxidation is at least a two-stage process. It seems that the most likely explanation of this effect is the aforementioned (referenced in the Introduction) decomposition of the OAlO complex formed in the first stage of aluminum oxidation to AlO with oxygen released simultaneously (to the atmosphere above the sample). 

The oxidation of aluminum powder was problematic due to the low melting-point (660.3 °C) and the fast sublimation of Al particles. The XRD pattern of oxidized aluminum powder did not confirm the presence of oxide (Al_2_O_3_). This observation is consistent with the literature where amorphous Al_2_O_3_ is featured [37,38]. In the case of aluminum powder oxidation, amorphous Al_2_O_3_ is thermodynamically more stable than the crystalline form. The amorphous Al_2_O_3_ oxide is very thin, and its growth is controlled by the outward diffusion of aluminum cations that prevents crystallization of the oxide. Moreover, the further oxidation of aluminum is limited because of well-known protective properties of Al_2_O_3_ [39]. At the temperature higher than 300 °C, the amorphous Al_2_O_3_ layer reaches the critical thickness which ranges between 0.5 nm and 4 nm depending on the study [40,41] and after that crystalline γ-Al_2_O_3_ begin to form. Around the temperature 800 °C, the γ-Al_2_O_3_/θ–Al_2_O_3_ scale is stable. In the temperature-range 850–1100 °C, θ-Al_2_O_3_ transforms to α-Al_2_O_3_ [42,43]. Even at the temperature 1400 °C, the complete oxidation of the aluminum powder did not take place [43].

Aluminum powder reduction was not carried out to very high-temperatures due to the possibility of aluminum evaporation. Al_2_O_3_ in contact with aluminum can be reduced according to the reaction written in the introduction part to the aluminum nano-powder, which can very easily evaporate. It was also possible that nanosized aluminum from the reduction of Al_2_O_3_ can further react with hydrogen with the formation of aluminum hydride (AlH_3_), which can then very-readily decompose. According to the phase diagram calculated from Barin and Knacke data [44], aluminum from alumina can be obtained at a temperature much higher than the melting-point of aluminum, and, at the same time, the partial pressure of the water vapor should be kept very low [45]. On the other hand, hydrogen can dissolve in molten aluminum and the amount of dissolved hydrogen can be calculated with the empirical equation [46]:

log(S/S°) − 1/2log(p/p°) = (−2700/T) + 2 · 72(7)

where: S° is a standard value of solubility equal to 1 cm^3^ of diatomic hydrogen measured at 0 °C and 101325 Pa (1 atm) per 100 g of metal, p° is a standard pressure equal to 101325 Pa (1 atm), and T is absolute temperature. It is generally accepted that hydrogen is dissociated into the atomic state when dissolved in aluminum which can explain possibility of alumina reduction when alumina is in contact with molten aluminum. A certain amount of molecular hydrogen is probably also present in hydrogen gas [45]. It was experimentally confirmed [45] that it is possible to reduce alumina with hydrogen to aluminum when molten aluminum is present in the system. However, at the same time competitive reaction can take place:

2Al_(l)_ + H_2(g)_ ⇆ 2AlH_(g)_ (8)

Al_2_O_3(s)_ + 6AlH_(g)_ ⇆ 8Al_(l)_ + 3H_2_O_(g)_ (9)

At the temperature below the melting-point of aluminum (450–550 °C) nonstoichiometric black alumina (Al_2_O_2.908_) was detected after hydrogen reduction [47]. The amount of nonstoichiometric alumina was consistent with possible surface reduction:

Al_2_O_3(surf)_ + H_2(g)_ = 2AlO_(surf)_ + H_2_O_(g)_ (10)

In the case of the Ti–Al powder alloy, the TPOx profile in the temperature-range 550–750 °C is similar to the aluminum profile in the temperature-range 230–430 °C, as can be seen in Figure 1a. Therefore, it can be concluded that the decomposition of the OAlO complex for Ti–Al powder is visible at a temperature around 300 °C higher than for pure aluminum powder. Furthermore, it is worth noting that around 270 °C slight oxygen consumption begins, which can confirm the formation of the Al_2_O_3_ oxide layer. However, it seems that the oxide layer was very thin or amorphous because in the case of Ti–Al powder after TPOx conducted up to 530 °C and intentionally interrupted for XRD analysis (not presented here) oxides were not detected. The only recognized phases according to XRD came from the substrate powder: Ti–Al (the main phase) and Ti_3_Al (the minor phase). 

Additionally, as shown in Figure 1a on the Ti–Al powder TPOx profile, around 750 °C the main oxidation process begins, which is probably connected with titanium oxidation, since the shape of the TPOx profile is very similar to this measured for titanium powder but at around 300 °C lower-temperature. The results obtained for the Ti–Al powder confirmed the higher oxidation resistance of the Ti–Al alloys described in the literature compared to those of pure titanium and aluminum. 

The TPR profile for Ti–Al powder after TPOx shows the maximum shifted to the higher-temperature (beginning around 460 °C) in comparison to the maximum that appeared for titanium reduction. The effect is small compared to that seen for pure titanium. 

It is worth noting that in the case of oxidation and reduction of Ti–Al material on TPOx/TPR profiles, slight effects related to the reaction occur, while the phase composition determined on the basis of XRD measurements does not change. This fact proves that the TPOx/TPR method is much more sensitive compared to the XRD method.

Analysis of the behavior of the niobium powder sample during thermal heating in oxidizing atmosphere shows that the first oxidation effect appears for Nb at about 220 °C (Figure 1a). The shape of the TPOx profile in Figure 1a corresponding to the oxidation of niobium proves that it is a quite rapid process which consists of at least three stages. The first peak (composed of two maxima at 380 and 430 °C) probably corresponds to oxidation of niobium to lower oxides, the next effect (at about 460 °C) is inhibition of the oxidation process, and the last one is the next peak corresponding to the oxidation process. From around 480 °C, a decrease in the TPOx line, characteristic of the so-called “oxygen starvation”, can be seen similar to the effect for titanium powder.

**Figure 4:**
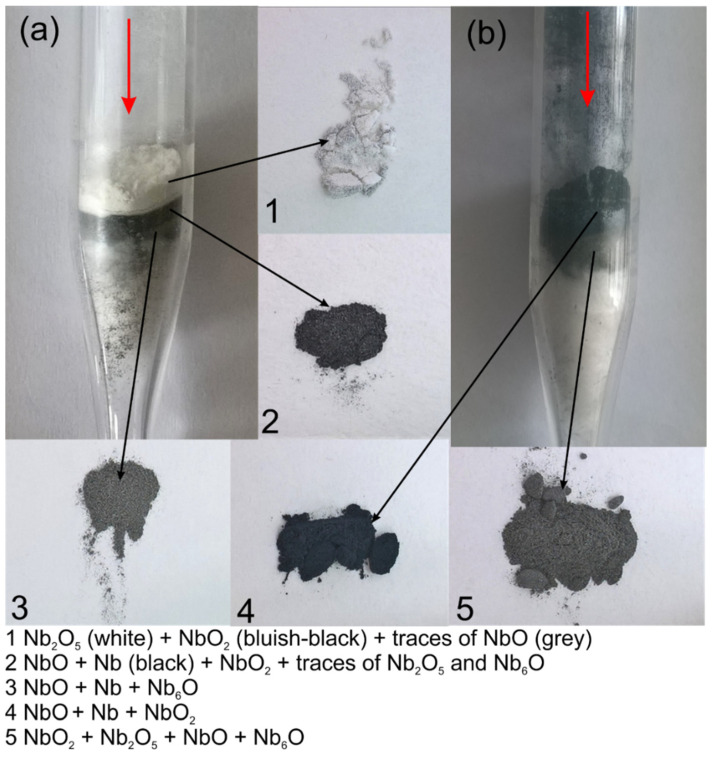
Quartz reactor with Nb powder after TPOx (**a**) and after TPOx + TPR (**b**) and the powder fractions marked as 1–5 with corresponding possible phases revealed with XRD. Arrows indicate the flow direction of the O_2_/Ar or H_2_/Ar mixture.

The three stages of oxidation visible in the TPOx line for niobium correspond to the three layers of niobium oxide reported in [12] and with the three main layers of niobium powder visible in the digital picture of the reactor in Figure 4a. All the visible layers of powder were analyzed with XRD separately. 

**Figure 5:**
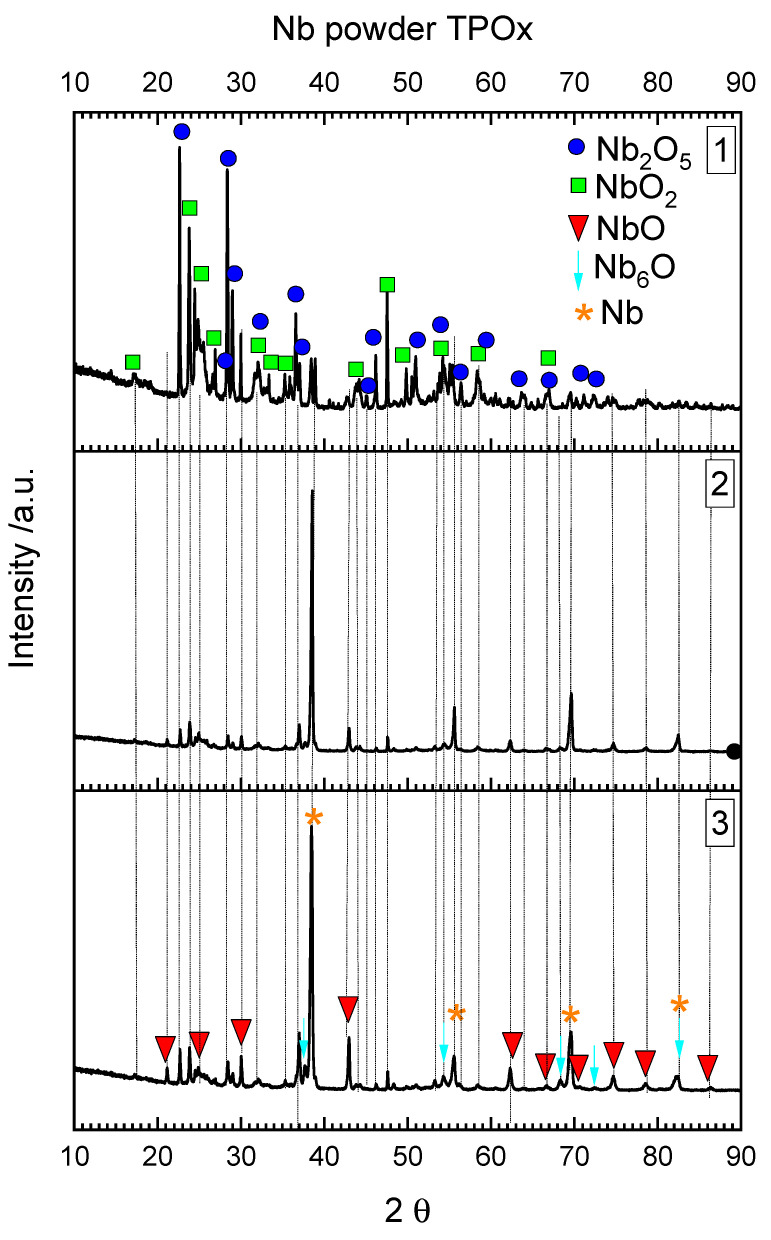
XRD diffraction pattern from the Nb powder after TPOx (the numbers in the upper right corner are related to the powder fractions marked in Figure 4).

The outermost white layer, which was in contact with the fresh O_2_/Ar gas mixture during TPOx, is mainly composed of Nb_2_O_5_ (Figure 5). Some lower oxides such as NbO_2_ or NbO were also detected. The middle, dark layer contained mainly NbO (Figure 5) and traces of lower oxides. The lowest layer was dark grey and was also composed of NbO but with some amount of unoxidized niobium powder. In the middle and in the lowest layer, Nb_6_O was also detected. Nb_6_O is a nonstochiometric niobium oxide. 

In the literature between Nb and NbO, three metastable niobium oxides were described and were classified as NbO_x_, NbO_y+_, and NbO_z_. NbO_x_ with a stoichiometry equivalent to Nb_6_O was reported to be formed at 270–530 °C. NbO_y_ with stoichiometry equivalent to Nb_4_O was created at 330–500 °C and NbO_z_ with unknown stoichiometry was formed at 400–700 °C [48]. Nb_6_O was also detected in a scale formed during the annealing of Nb_40_Ti_30_Ni_30_ alloy in air at elevated-temperature [49]. The other authors claim that the first crystalline phase formed during the niobium oxidation is Nb_6_O; however, the earlier appearance of the amorphous phase hinders the formation of this oxide [50]. Niobium oxides are also considered as catalysts for hydrogen adsorption or desorption processes. Hanada et al. [51] claimed that NbO instead of Nb_2_O_5_ is responsible for the catalytic activity of niobium oxides and, moreover, Takahashi et al. [52] confirmed this effect on the basis of the DFT study.

In the TPOx experiment presented here, all possible niobium oxides were recognized with increasing oxygen content in the upper layer direction which was in contact with the oxygen-rich gas. 

As can be seen in the TPR line in Figure 1b, the Nb powder was reduced from approximately 360 °C, close to the temperature at which the main effect of Ti reduction also started. The shape of the TPR profile indicates that the reduction was gradual and, therefore, was most likely related to the reduction of several oxide species. It is also visible that the reduction of niobium oxides is not very intense. The measurement presented in [53] made by the temperature-programmed reduction method, carried out at a heating rate of 10deg/min, indicates that the reduction of Nb_2_O_5_ starts at about 350 °C, while the maximum reaches around 800 °C which is in accordance with our results. 

**Figure 6:**
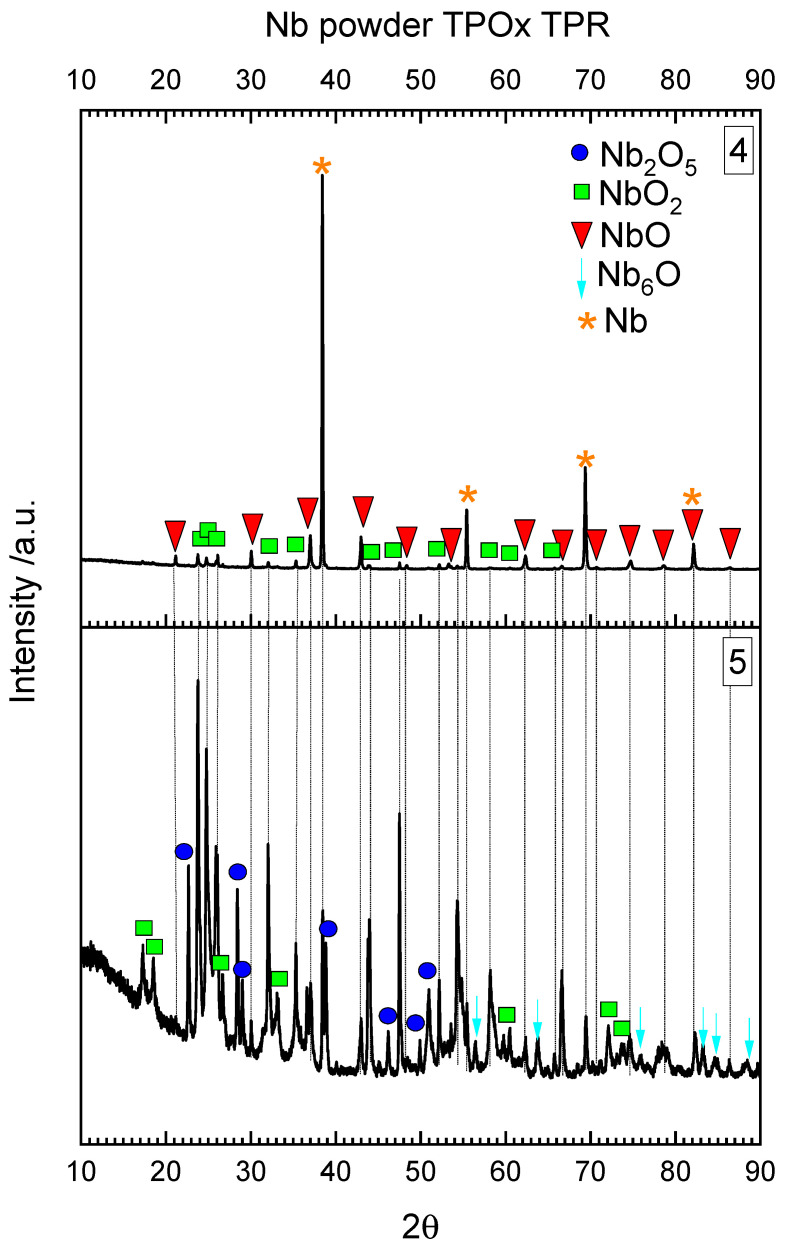
XRD diffraction pattern from the Nb powder after TPOx and TPR (numbers in the upper right corner are related to the powder fractions marked in Figure 4).

After reduction (followed by the oxidation process) the two main powder layers are visible in the digital picture of reactor in Figure 4b. Both layers were analyzed separately with XRD (Figure 6). As follows from Figure 6, the upper layer was the most reduced (this layer was in contact with fresh H_2_/Ar mixture) and composed with NbO_2_ and NbO and some amount of metallic niobium powder. The underneath powder layer still contained the highest niobium oxide (Nb_2_O_5_), which was not reduced, probably due to the insufficient amount of hydrogen in the gas mixture. It is also difficult to recognize which of the lower niobium oxides were produced during reduction (TPR), and which of them remained after incomplete oxidation. The results presented here confirmed that reduction of Nb_2_O_5_ into the metallic niobium is not an easy process and probably requires stronger reducing atmospheres, as was mentioned in the introduction part. On the basis of the presented results, it could be seen that the oxidation and reduction of niobium are complex phenomena. Further investigations are planned to better understand the mechanisms of niobium oxidation and reduction. 

### 3.2. Oxidation and Reduction of Solid Samples

**Figure 7:**
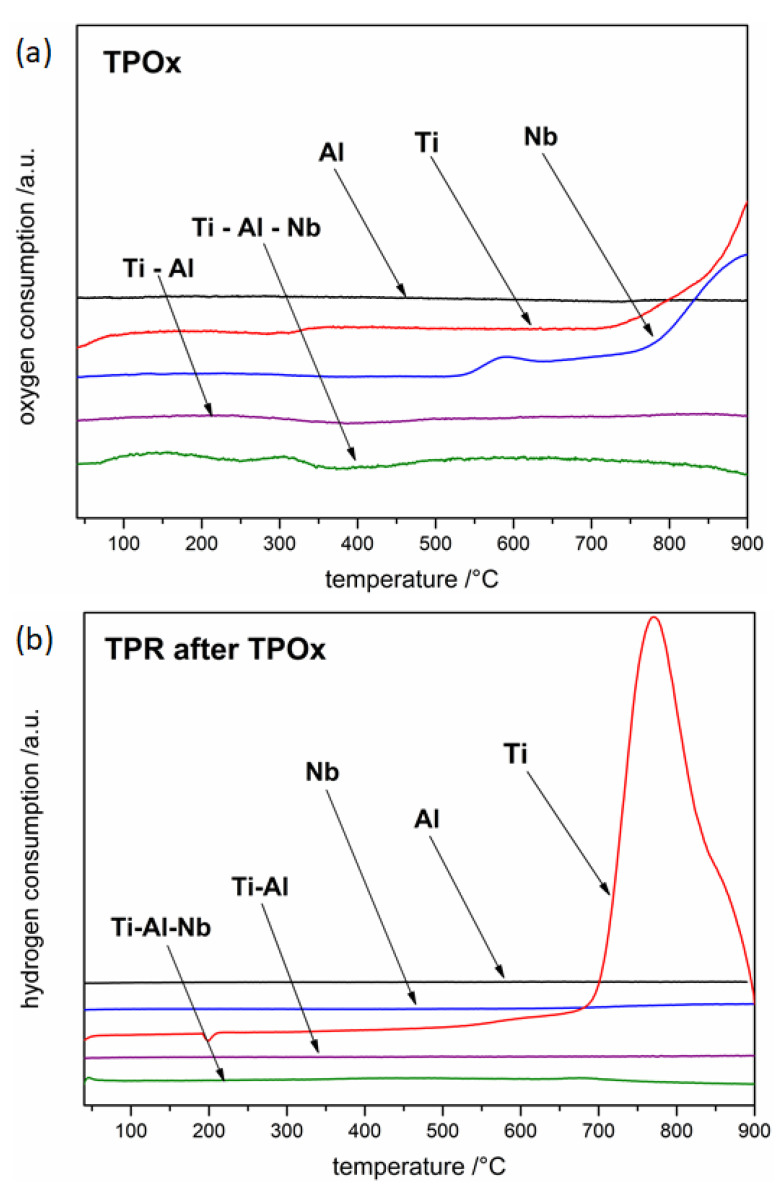
The oxidation (**a**) and reduction (**b**) profiles of tested solid samples.

TPOx and TPR profiles (Figure 7) of solid samples are the basis for considerations regarding corrosion processes (in an atmosphere containing oxygen and containing hydrogen) of the tested metals and their alloys. Figure 7 clearly shows that both the oxidation and reduction processes, in the case of all tested materials in the form of solid samples, took place at a higher-temperature than for proper powder materials. The TPOx profiles indicate that the Ti sample oxidized at the lowest-temperature (the beginning around 320 °C, but the main oxidation at 720 °C), the next was the Nb sample (beginning at 520 °C), and only the Al sample in the all-controlled temperature-range did not oxidize. Moreover, in the case of the aluminum sample, the oxygen-releasing effect that most likely occurred on the powder material, was not observed. It can be suspected that this is the result of the small surface area of the solid sample compared to the area of powder, and, thus, the effect related to the formation and decomposition of the OAlO complex is imperceptible. Importantly, the Ti–Al alloy at the tested temperature-range did not undergo the oxidation process, which proves that this material was much more resistant to oxygen corrosion compared to pure titanium. The TPR of the Ti–Al alloy profile does not indicate any effects of hydrogen consumption, which additionally confirms that the material was resistant to high-temperature corrosion in the presence of oxygen. In order to check the influence of niobium on the Ti–Al corrosion behavior, Ti–Al–Nb solid sample was also investigated. As mentioned in the introduction, an increase in corrosion resistance was expected. As can be seen in TPOx and TPR profiles, similarly to the Ti–Al alloy, the Ti–Al–Nb material exhibited high resistance to high-temperature oxygen and hydrogen corrosion.

However, the result of diffraction analysis carried out for solids (after oxidation, and reduction) indicates that their compositions change in relation to the compositions of the starting materials, even if the TPOx/TPR profiles do not indicate the occurrence of the reaction.

**Figure 8:**
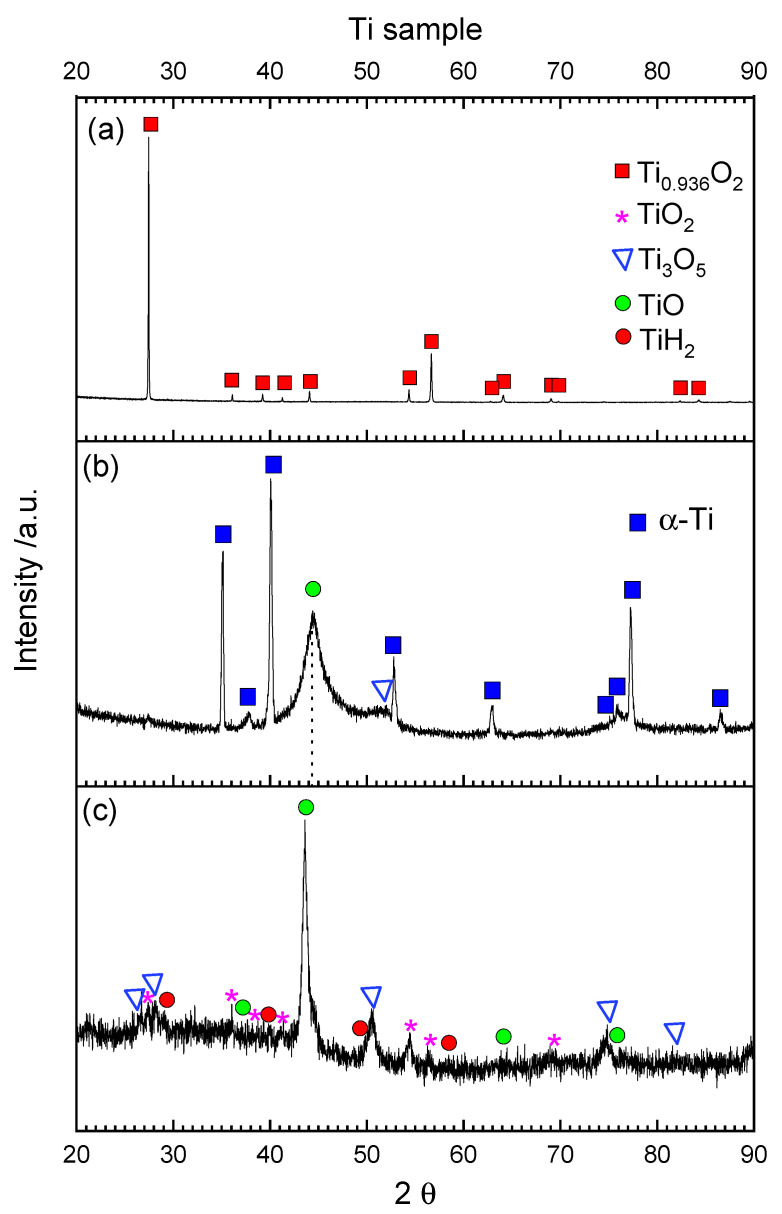
XRD diffraction pattern of the Ti solid sample after TPOx (spalled scale (**a**), sample surface below the scale, XRD-GID (**b**)) and TPOx + TPR (surface of sample, XRD-GID (**c**)).

As can be seen from the diffraction pattern in Figure 8a, the oxidation of the titanium sample was slightly different than the oxidation of the titanium powder. At the surface of oxidized sample only Ti_0.936_O_2_ rutile phase was detected. According to the literature Ti_0.936_O_2_ (anatase) has very good photocatalytic performance [54]. This phase was also detected on the surface of Ti–6Al–4V after electrochemical corrosion test in the LiF–NaF–KF molten salts mixture at 550 °C [55]. In Figure 8b, XRD analysis for Ti sample after oxidation and scale spallation is presented. The wide reflex between 40 and 50° probably indicated some lower oxides, e.g., TiO under the thick Ti_0.936_O_2_ layer. In contrast to Ti powder reduction, the composition of the solid sample after TPR was more complex, probably due to incomplete reduction. As can be seen in (Figure 8c), the TiO_2_ residues are accompanied with two lower oxides: TiO and Ti_3_O_5_. Moreover, some very small amount of undecomposed TiH_2_ was also identified with XRD analysis. 

**Figure 9:**
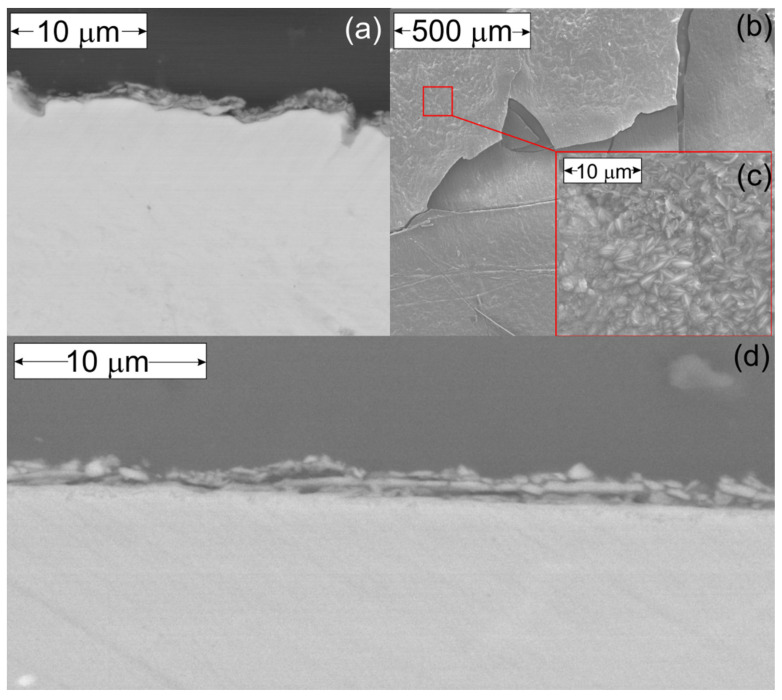
BE image of Ti sample cross-section after TPOx without the spalled scale (**a**) and SE image of the spalled scale (**b**) with visible rutile crystals in the higher-magnification frame (**c**). BE image of Ti–Al–Nb sample cross-section after TPOx + TPR (**d**).

In comparison to the composition of powder after reduction (Figure 3), the titanium sample reduced to a lesser extent. It is also possible that the detected TiO and Ti_3_O_5_ oxides were present before reduction after oxidation beneath TiO_2_ and was exposed after TiO_2_ spallation. TiO_2_ spallation was caused by the temperature changes during cooling and heating between TPOx and TPR measurement. It is well known that the thermal expansion coefficients for titanium and TiO_2_ (rutile) are different and equal, respectively: 8.5 · 10^−6^/deg (at RT) and depending on the rutile unit cell crystallographic axes a = 6.99953 · 10^−6^/deg, c = 9.36625 · 10^−6^/deg, and = 28.680 10^−6^/deg, which can promote scale spallation under thermal cycling conditions [56,57]. This hypothesis is also in agreement with the morphology of scale formed on titanium after oxidation described in the introduction part and with the microscopic observations of titanium cross-section and scale after oxidation presented in Figure 9a–c. As it is visible the spalled scale was rather thick with a well-crystallized rutile phase. The thickness of the oxide layer remaining on the surface was about 1.5 µm.

**Figure 10:**
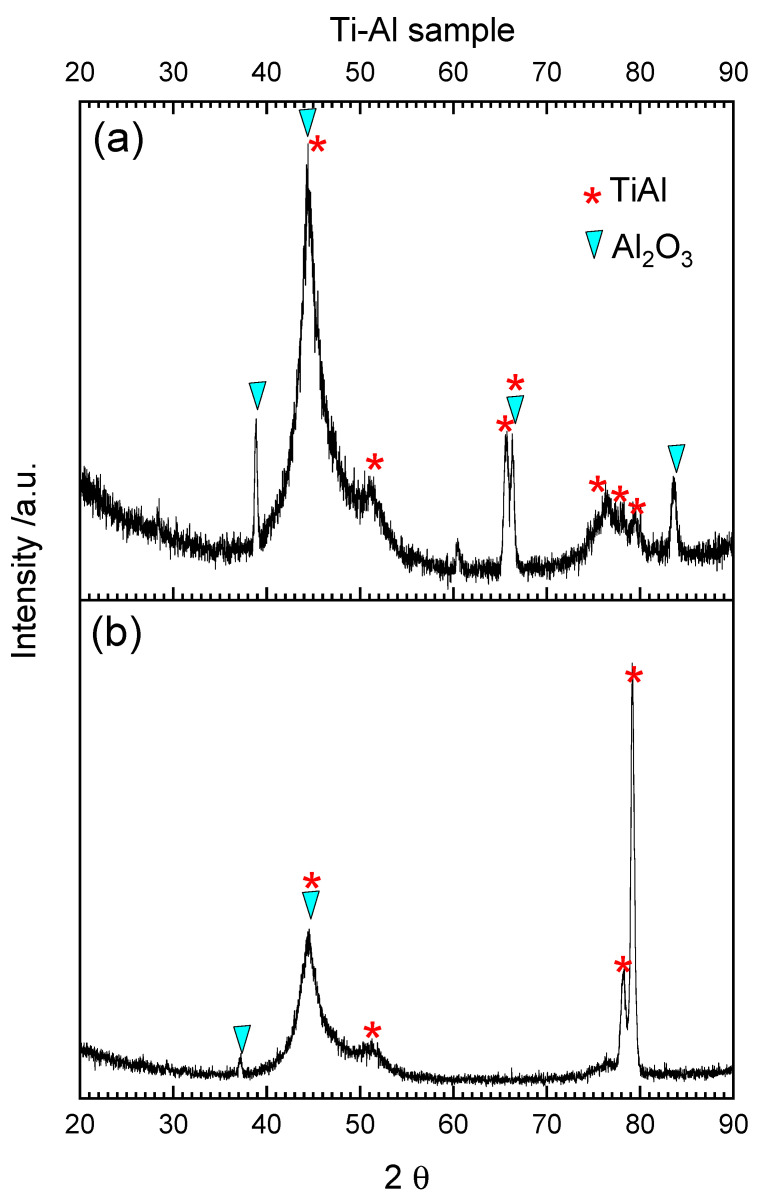
XRD-GID diffraction pattern from the Ti–Al solid sample after TPOx (**a**) and TPOx + TPR (**b**).

XRD results for the Ti–Al sample are shown in Figure 10a. The broad peaks indicate the presence of amorphous phase, probably Al_2_O_3_. Some reflexes can be identified as -Al_2_O_3_ (with exact stoichiometry Al_2.666_O_3.999_). The main alloy phase (TiAl) can also be recognized. After reduction the amount of aluminum oxide is lower and some reflexes from the Ti–Al phase became more visible (Figure 10b). These results are not in accordance with the literature, because thermodynamical calculations showed that for titanium aluminides, containing about 50 at.% aluminum or less, titanium oxide rather than aluminum oxide was the stable phase [58]. In other articles, it was found that approximately 60–70% of aluminum is needed for Ti–Al alloys to form a continuous layer of Al_2_O_3_ during oxidation in air and approximately 47–49% of aluminum is needed during oxidation in pure oxygen [59,60,61].

**Figure 11:**
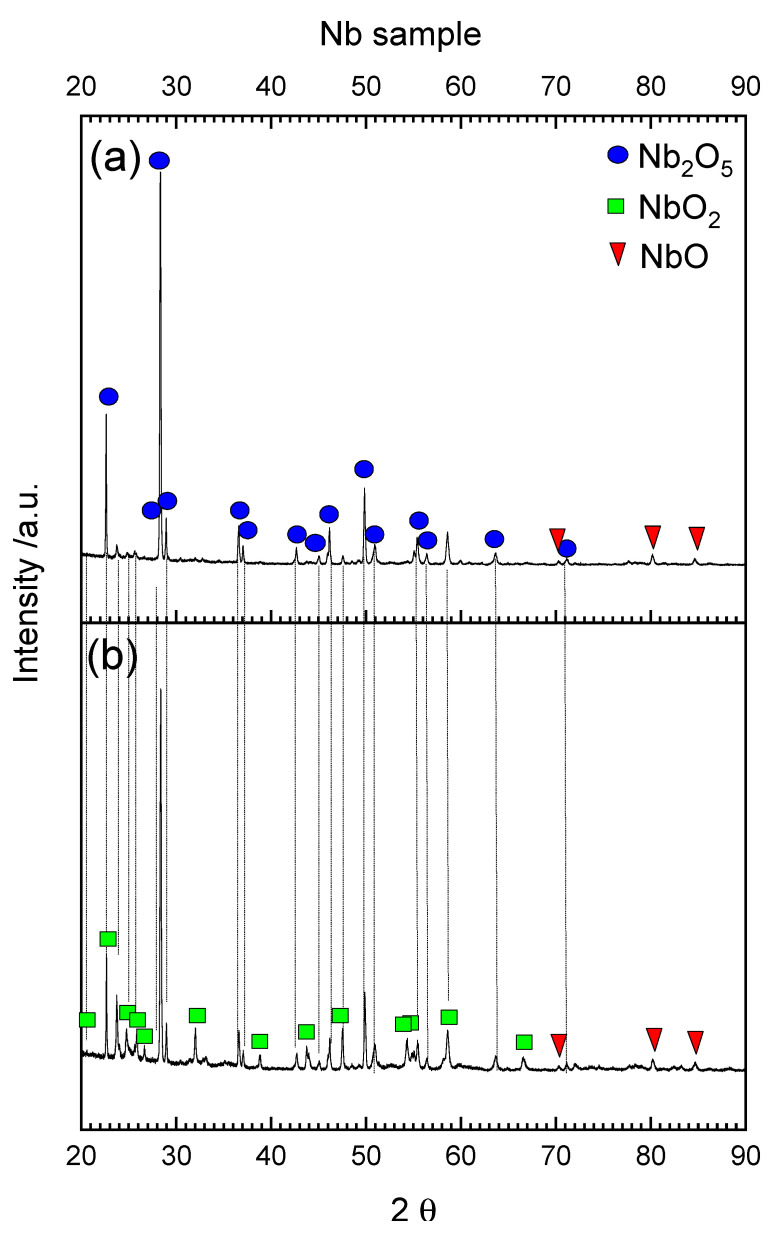
XRD diffraction pattern from the Nb solid sample spalled scale after TPOx (**a**) and TPOx + TPR (**b**).

The composition of the scale formed on the niobium sample after oxidation is very simple and the highest oxide was mainly detected (Figure 11a). Niobium has a high affinity for oxygen and can very easily create Nb_2_O_5_ oxide. The presence of lower niobium oxides is possible only when the insufficient amount of oxygen is used [62]. It is also possible that beneath Nb_2_O_5_ in the oxide/metal interface some lower Nb oxides were present, as visible in Figure 11a NbO_2_ and NbO. After reduction (Figure 11b) the scale was built mainly with niobium dioxide (NbO_2_) probably from Nb_2_O_5_ reduction and with a smaller amount of Nb_2_O_5_ and NbO. Oxidation and reduction of a solid sample similar to titanium is different than for powder. In the case of powder, the surface in contact with the oxidizing/reducing agent is large and the oxidation/reduction is faster, limited only by the availability of the reactant. In the case of solid samples, the surface is relatively small and the products created at the surface can behave as a diffusion barrier for further oxidation/reduction. 

**Figure 12:**
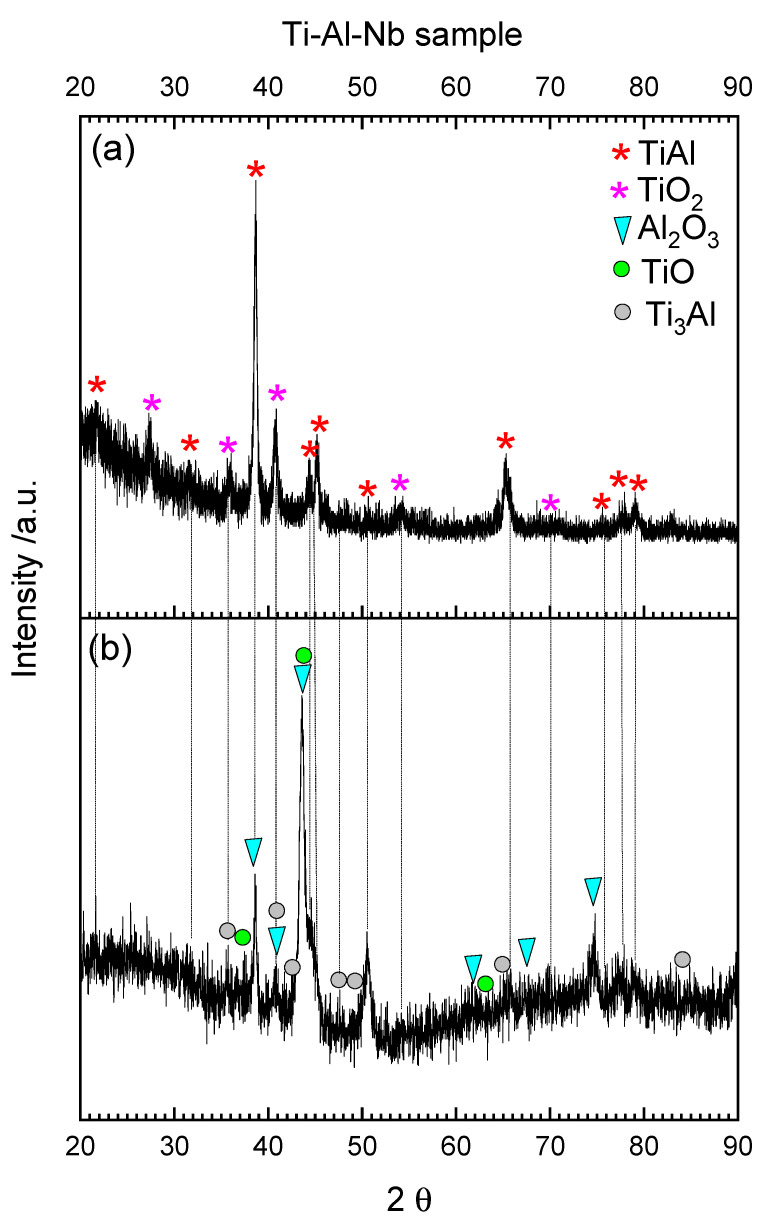
XRD-GID diffraction pattern from the Ti-Al-Nb solid sample after TPOx (**a**) and TPOx + TPR (**b**).

Interpretation of XRD results for Ti–Al–Nb sample after oxidation and reduction is difficult because many reflexes overlapped. As can be seen in the XRD pattern in Figure 12a after oxidation only two phases are clearly visible: TiAl (substrate) and TiO_2_ (rutile). However, the presence of small amount of Al_2_O_3_ was also possible, because some reflexes of TiAl and Al_2_O_3_ phases overlapped. The presence of the second substrate alloy phase-Ti_3_Al after oxidation was not confirmed with XRD but it is not excluded because reflexes from this phase overlapped with those from TiO_2_. According to the literature at the beginning of oxidation, oxygen dissolves in the alloy and the amount of dissolved oxygen depends on the TiAl phase and is equal: 16 at.% for α_2_-Ti_3_Al and 2 at.% for TiAl [63]. Next ultrathin layer of alumina is formed (about two times thicker for α_2_-Ti_3_Al) which results in Al-depletion in metallic phase. When the critical concentration of aluminum is reached, titanium oxidation occurs and TiO_2_ is formed on the surface. 

According to the diffraction pattern in Figure 12b after TPR, the outermost layer of TiO_2_ was reduced to TiO. The inner layer of Al_2_O_3_ became more visible and was not completely reduced. Reflexes from the Ti_3_Al phase were easier to identify in the XRD pattern after reduction. The presence of very thin (around 0.5 μm) remaining oxide scale after TPR was confirmed with microscopic observations shown in Figure 9d. 

Note that in the case of all tested solid samples, both the phase composition (XRD) and the observations with SEM give more information than the TPOx/TPR method, which was definitely more useful in the case of powder samples. 

## 4. Conclusions

Titanium aluminides are interesting for structural applications in transportation, because they have low density, high strength, and promising resistance to oxidation at moderate-temperatures. Therefore, in-depth analysis of the oxidation and reduction processes of alloys is particularly important in the context of their resistance to the oxidizing and reducing atmosphere. The oxidation and reduction of powder materials based on the Ti–Al system are also important for catalytic applications, because titanium and aluminum oxides are often used as supports for catalytically active metals in redox reactions. Additionally, some authors consider niobium oxides as catalysts for hydrogen adsorption or desorption processes.

The mechanisms of Al, Ti, Nb, Ti–Al, and Ti–Al–Nb oxidation and subsequent reduction were different for powders and solid samples what was confirmed by TPOx/TPR profiles, as well as by phase composition analysis and microscopy observations after oxidation and reduction processes. In the next experiments it is planned to recognize if the differences between oxidation and reduction of powders and solids are kinetic or thermodynamic in nature. Further investigations are also necessary to specify the exact mechanism for Ti–Al–Nb alloy oxidation. Especially understanding of the alloy reduction may be of great importance in the context of its predicted application in the aerospace or automotive industry (construction of valves, turbocharger rotors, and components of exhaust systems), where hydrogen fuel is the future.

The TPOx results showed that the Ti–Al sample is more resistant to oxidation than titanium and aluminum separately, both for powder materials and solid samples. The oxidation of Ti–Al is often described in the literature as a two-stage process, whereas hereby three stages of oxidation were revealed: the first stage was the formation and decomposition of the OAlO complex, the next creation of amorphous Al_2_O_3_, followed by the growth of TiO_2_. 

It seems that the introduction of niobium into the Ti–Al system changed the mechanism of Ti–Al–Nb alloy oxidation. Interestingly, in the case of Ti–Al–Nb oxidation, TiO_2_ was the predominant phase, while alumina was probably only in the amorphous form. This result is in contradiction to the beneficial effect of niobium in alumina formation described in the literature. Detailed explanation of the influence of niobium on the oxidation, as well as the reduction of the Ti–Al–Nb alloy is planned in the future. Well-controlled TPOx/TPR conditions and in situ XRD measurement are considered. 

The factor significantly affecting the usefulness of the TPOx/TPR method is the specific surface area of the tested materials; in the case of powder materials, the sensitivity of these methods was greater than that of the X-ray diffraction method, hence its use to study the reaction mechanism was fully justified. It was different in the case of testing solid materials, when this method was definitely less useful due to small specific surfaces of the samples. Moreover, TPOx/TPR allows to investigate only relatively short-term processes. 

## Data Availability

Data will be made available upon reasonable request.
